# Safety analysis of self-administered enzyme replacement therapy using data from the Fabry Outcome and Gaucher Outcome Surveys

**DOI:** 10.1186/s13023-024-03416-2

**Published:** 2025-03-28

**Authors:** Shoshana Revel-Vilk, Uma Ramaswami, Guillem Pintos-Morell, Derralynn Hughes, Kathy Nicholls, Ricardo Reisin, Roberto Giugliani, Ozlem Goker-Alpan, Majdolen Istaiti, Aidan Gill, Maurizio Scarpa, Jaco Botha

**Affiliations:** 1 Gaucher Unit and Pediatric Hematology/Oncology Unit, The Eisenberg R&D Authority, Shaare Zedek Medical Center, Jerusalem, Israel; 2 Faculty of Medicine, Hebrew University, Jerusalem, Israel; 3 Lysosomal Storage Disorders Unit, Royal Free London NHS Foundation Trust and University College London, London, UK; 4 Vall d’Hebron Institute of Research (VHIR), Vall d’Hebron Barcelona Hospital Campus, MPS-Spain Medical Committee, Barcelona, Spain; 5 Lysosomal Disorders Unit, Royal Free London NHS Foundation Trust, University College London, London, UK; 6 The Royal Melbourne Hospital, University of Melbourne, Parkville, VIC Australia; 7 Hospital Británico de Buenos Aires, Buenos Aires, Argentina; 8 Department of Genetics, UFRGS, Porto Alegre, Brazil; 9 Dasa Genomics, Sao Paulo, Brazil; 10 Casa dos Raros, Porto Alegre, Brazil; 11 INAGEMP, UFRGS, Porto Alegre, Brazil; 12 Medical Genetics Service, HCPA, Porto Alegre, Brazil; 13 Lysosomal and Rare Disorders Research and Treatment Center, Fairfax, VA USA; 14 Gaucher Unit The Eisenberg R&D Authority, Shaare Zedek Medical Center, Jerusalem, Israel; 15 Takeda Development Center Americas (at time of study start), Lexington, MA USA; 16 Regional Center for Rare Diseases, University Hospital of Udine, Udine, Italy; 17 Takeda Pharmaceuticals International AG, Zurich, Switzerland

**Keywords:** Enzyme replacement therapy, Self-administration, Agalsidase alfa, Velaglucerase alfa, Safety, Fabry, Gaucher, FOS, GOS, Home therapy

## Abstract

**Background:**

Fabry disease and Gaucher disease are rare genetic disorders characterized by defective degradation of glycosphingolipids caused by enzymatic deficiencies in α–galactosidase A and β–glucocerebrosidase, respectively, and often require life-long treatment. Treatment options for these disorders include replacing the deficient enzymes via enzyme replacement therapy (ERT). Agalsidase alfa for Fabry disease and velaglucerase alfa for Gaucher disease are two ERT options with demonstrated efficacy, safety, and tolerability. ERT infusions administered by a health care provider (HCP) in the clinic/hospital, or at the patient’s home are considered HCP-supported infusions. Self-administration of ERT (by patient, partner, relative, or caregiver) is optional in patients who tolerate the HCP-supported infusions at home and have a suitable home environment. This analysis explored the safety profiles of self-administered agalsidase alfa (202 patients) and velaglucerase alfa (30 patients) versus HCP-supported infusions using data from the Fabry Outcome Survey (FOS) and Gaucher Outcome Survey (GOS) registries.

**Results:**

The frequency of infusion-related reactions (IRRs) adverse events (AEs) recorded in the two registries was lower in patients self-administering (FOS: 4.5%, GOS: 0%) versus patients receiving HCP-supported infusions (FOS: 13.6%, GOS: 1.6%). In the FOS registry, AE rates per 100 patient-years (100PY) of follow-up were similar between the self-administration (7.99) and HCP-supported infusion (6.78) groups. In patients self-administering agalsidase alfa, cardiac disorders were the most frequently reported AEs (19 [9.4%] patients) and serious AEs (12 [5.9%]) and gastrointestinal disorders were the most frequently reported IRRs (3 [1.5%]). In the GOS registry, AE rates per 100PY were similar between self-administration (4.97) and HCP-supported infusion (4.67) groups. In patients self-administering velaglucerase alfa, skin and subcutaneous disorders (4 [13.3%]) and infections and infestations (2 [6.7%]) were the most reported AEs and serious AEs, respectively, and no IRRs were reported.

**Conclusions:**

These findings suggest that self-administration of agalsidase alfa or velaglucerase alfa infusions are not associated with additional safety risks compared with HCP-supported infusions and are a suitable option for qualifying patients. Further research is warranted to support these findings and to explore further the long-term safety and efficacy of ERT self-administration.

FOS trial registration: ClinicalTrials.gov, NCT03289065. Registered 01 April 2001, https://clinicaltrials.gov/study/NCT03289065. GOS trial registration: ClinicalTrials.gov, NCT03291223. Registered 27 July 2010, https://classic.clinicaltrials.gov/ct2/show/NCT03291223.

## Background

Fabry disease and Gaucher disease are rare genetic lysosomal storage disorders characterized by defective degradation of glycosphingolipids due to enzymatic deficiencies in α–galactosidase A and β–glucocerebrosidase, respectively [1, 2]. Patients with Fabry disease suffer from progressive damage to renal, cardiac, and central nervous systems, and report recurrent episodes of neuropathic pain and gastrointestinal disturbance [3, 4]. There are two recognized forms of Fabry disease (classical and late-onset). Classical Fabry disease usually starts in childhood or adolescence with symptoms occurring earlier in males compared with females [5, 6]. Renal, cardiovascular, or cerebrovascular complications may lead to premature death in patients with untreated classical Fabry disease [3, 7]. Late-onset Fabry disease generally starts after age 30 and has a more variable disease course that is less severe, with disease manifestations sometimes limited to a single organ [8].

Symptoms of Gaucher disease include anemia, thrombocytopenia, hepatosplenomegaly, and skeletal complications [9], with age of onset and life expectancy varying according to disease type and severity. Type I, the most common form in Europe and the United States, is not associated with central neurological impairment, except for the predisposition of these patients to develop Parkinson’s disease, and may present at any stage of life [10, 11]. Types II and III are associated with neurological abnormalities, which are more severe in type II [11]. Type II regularly presents before the age of 6 months and is often fatal in infancy [11], while type III typically presents prior to age 20 with life expectancy ranging from adolescence to the fifth decade of life [11, 12].

Currently available treatment options for Fabry disease include three enzyme replacement therapy (ERT) agents (agalsidase alfa [not available in the United States], agalsidase beta, and pegunigalsidase alfa) and one oral pharmacological chaperone (migalastat, only for patients with amenable mutations) [13, 14]. For Gaucher disease, three ERTs (imiglucerase, velaglucerase alfa, and taliglucerase alfa [not available in the European Union]) and two oral substrate reduction therapies (miglustat and eliglustat) are available [15]. For patients with clinically relevant manifestations of Fabry or Gaucher disease, management is focused on replacing the deficient enzymes via ERT, oral pharmacological chaperone therapy (Fabry) or substrate reduction therapy (Gaucher). ERT has demonstrated efficacy through control of symptom progression, and acceptable safety and tolerability profiles in real-world studies for both Fabry [16, 17] and Gaucher [18, 19] diseases. In many countries, initial ERT infusions typically occur in a clinical/hospital setting and, once initiated, may be life-long [20, 21]. While ERT is effective, it may not address all disease manifestations in part due to the pharmacodynamics and biodistribution of enzymes [22], and in some cases where manifestations were already irreversible at the start of therapy.

ERT infusions can be administered by a health care provider (HCP) in the clinic/hospital, as described, or at the patient’s home, both of which are considered HCP-supported infusions. Home infusion by an HCP of agalsidase alfa (for Fabry disease) [23, 24] or velaglucerase alfa (for Gaucher disease) [9, 25] is an established option in some countries for patients who tolerate it and have a suitable home environment. Factors for establishing home infusions can include, but are not limited to, completion of a required number of in-clinic/hospital infusions, no infusion-related reactions (IRRs), appropriate conditions for receiving and storing the medicinal product to infuse at home, and an overall safety and risk assessment by the HCP prior to initiating home treatment. Home infusions are defined as administration of the infusion to a patient at home and solely relate to the location for the infusion to be performed and do not refer to who administers the infusion. Home infusions of agalsidase alfa and velaglucerase alfa have been associated with improvements in patient satisfaction and quality of life (QoL), compared with infusions in a clinic/hospital setting [26–29], and with treatment adherence greater than 97% [30, 31]. Additionally, home infusion has the potential to reduce the risk of hospital acquired infection, including COVID-19 [32, 33].

**Fig. 1:**
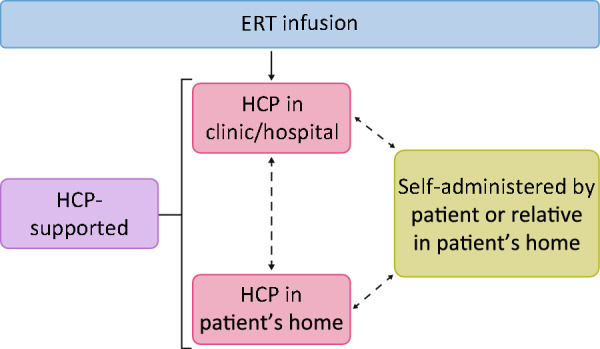
Patient transitions between HCP-supported and self-administered ERT infusions. In HCP-supported infusions, patients receive ERT infusions administered by an HCP in the clinic, hospital, or in the patient’s home. Patients can then transition to self-administering ERT infusions where either the patient (self), partner, relative, or caregiver administers the infusion in the patient’s home. Bidirectional dashed arrows indicate that a patient may transition from one therapy option to another, especially when an adverse event is experienced, and most patients return to the clinic/hospital for their next infusion following an adverse event. ERT, enzyme replacement therapy; HCP, health care provider

Patients tolerating infusions administered by an HCP at home may be eligible to self-administer treatment, or have treatment administered by their partners/relatives, with sufficient training [24, 28]. This training typically includes good hygiene practices, safe reconstitution, cannulation, administration, and disposal of ERT as observed by an HCP [24, 25, 28]. Self-administration is defined as administration of the ERT infusion via intravenous cannula either by the patient (self), parent, relative, or caregiver under the supervision of a physician (who may not be present on site). Self-administration can be performed by using a butterfly needle to access peripheral veins, or if needed, by using a fully implanted venous device (e.g., port-a-cath) [24, 28]. Patients are required to report any adverse reaction that may occur during or after each infusion [24, 28]. If the infusion is administered by the patient, a responsible adult must be present on-site and available to help in case of an emergency. The potential transition of patients between receiving HCP-supported ERT infusions to self-administering ERT infusions is shown in Fig. 1. Successful self-administration programs have been implemented for several conditions including multifocal motor neuropathy [34], hereditary angioedema [35, 36], and hemophilia [37] with no safety concerns in excess of those seen in the clinic setting. Due to the life-long nature of therapy for Fabry and Gaucher diseases, self-infusion at home may be desirable.

The objective of this analysis was to explore the safety profiles of agalsidase alfa and velaglucerase alfa when self-administered by the patients (or their partners/relatives) compared with HCP-supported infusions using data from the Fabry Outcome Survey (FOS) and Gaucher Outcome Survey (GOS) registries.

## Methods

### Study design and data sources

This international, multicenter, observational analysis examined patient data reported in the FOS (NCT03289065; https://clinicaltrials.gov/study/NCT03289065) [13] and GOS (NCT03291223; https://classic.clinicaltrials.gov/ct2/show/NCT03291223) [38] registries, sponsored by Shire Human Genetic Therapies, Inc., a Takeda company. The date of registration for FOS (NCT13289065) was April 1, 2001 and for GOS (NCT03291223) was July 27, 2010. The primary aims of the FOS and GOS registries are to provide long-term data on the effectiveness and safety of ERT for Fabry disease and Gaucher disease, respectively, broaden the understanding of each condition, and improve the clinical management of affected patients.

FOS and GOS registries have been approved by the ethics institutional review boards of participating centers and are conducted in compliance with relevant global and local regulations and best practices, Good Pharmacoepidemiological Practice, Good Research for Comparative Effectiveness principles, and the relevant principles of the International Conference on Harmonisation Good Clinical Practice guidelines, sixth edition (ICH E6), as appropriate for an observational registry. All participants provided written informed consent.

### Population

Patients enrolled in FOS or GOS with a confirmed diagnosis of Fabry disease or Gaucher disease who received ≥ 1 dose of study drug were eligible for inclusion. The self-administration populations included: patients enrolled in the FOS registry who self-administered ≥ 1 dose of agalsidase alfa with a confirmed Informed Consent Form (ICF) as of January 7, 2021; patients enrolled in the GOS registry who self-administered ≥ 1 dose of velaglucerase alfa with confirmed ICF as of July 30, 2021. Patients with missing start dates for self-administration were excluded from the analysis, while those with missing end dates were assumed to be continuing self-administered infusions, unless withdrawn from the registry.

Self-administration was defined as administration of ERT infusion via intravenous cannula either by the patient (self), partner, relative, or caregiver under the supervision of a physician (who may not be present on site) in the patient’s home. If the infusion was administered by the patient, a responsible adult was required to be present on-site and available to help in case of an emergency. Home infusion was defined as the administration of ERT infusion to a patient at home, was solely related to the location of the infusion, and did not refer to who administered the infusion. Patients who received home therapy with ERT infusions administered by an HCP regardless of location were not included in the self-administration populations. The populations who received HCP-supported infusions included patients enrolled in FOS or GOS who received ≥ 1 dose of study drug administered by HCP in the clinic, hospital, or at the patient’s home. The general practices of home therapy, self-administration, and approaches to patient training differed among the individual countries included in this analysis (Table 1).

**Table 1:** Home administration practices per country

Country	Home therapy availability	Self-administration allowed	Patient training
Argentina [23]	Available, first 3 infusions administered by HCP in hospital or infusion center, most home infusions administered by HCP	Yes, but not done as far as known for Fabry	N/A
Australia [40–42]	Available, organized by treating physicians for patients after a minimum of 12 agalsidase alfa or 3 velaglucerase alfa in-hospital infusions	Yes	N/A
Brazil [43]	Not available, but was allowed in some circumstances since COVID-19 pandemic	No	N/A
Canada [44]	Available with agalsidase alfa	Yes	N/A
Czech Republic [45, 46]	Available, encouraged during the COVID-19 pandemic. May be considered for patients who are tolerating their infusions well	Yes, for agalsidase alfa. Velaglucerase alfa application in process	Yes, given by treating physician and/or nurse to the patient/caregiver
Denmark [46]	Available, may be considered for patients who are tolerating their infusions well	Yes, for agalsidase alfa. Velaglucerase alfa application in process	Yes, given by treating physician and/or nurse to the patient/caregiver
France [46]	Available, may be considered for patients who are tolerating their infusions well	Yes, for agalsidase alfa. Velaglucerase alfa application in process	Yes, given by treating physician and/or nurse to the patient/caregiver
Germany [27, 46]	Available, patients must have uneventful ERT infusions in a hospital setting prior to initiating home therapy with infusions administered by HCP	Yes, for agalsidase alfa. Velaglucerase alfa application in process	Yes, given by treating physician and/or nurse to the patient/caregiver
Israel [25, 47]	Available, first 3 infusions administered by HCP in hospital or clinic	Yes	Yes, HCP educates on hygiene, reconstitution, cannulation, administration, and disposal
Italy [3, 46]	Available, administered by HCP and may be considered for patients who are tolerating their infusions well	Yes, for agalsidase alfa. Velaglucerase alfa application in process	Yes, given by treating physician and/or nurse to the patient/caregiver
The Netherlands [24, 46]	Available, patients must demonstrate accurate preparation and administration of the infusion in a hospital. If a significant reaction occurs at home, patients are required to return to the hospital for their next infusion	Yes, patients must perform 5 self-administered infusions at home during office hours in case assistance is required. Patients have various levels of nursing dependency with home therapy (independent, semi-independent, etc.)	Yes, HCP educates on hygiene, reconstitution, cannulation, administration, and disposal
Slovenia [46]	Available, may be considered for patients who are tolerating their infusions well	Yes, for agalsidase alfa. Velaglucerase alfa application in process	Yes, given by treating physician and/or nurse to the patient/caregiver
Spain [46]	Available for some patients with infusions administered by HCP; most infusions administered in a day-clinic setting	Yes, for agalsidase alfa. Velaglucerase alfa application in process	Yes, given by treating physician and/or nurse to the patient/caregiver
Sweden [46]	Available, may be considered for patients who are tolerating their infusions well	Yes, for agalsidase alfa. Velaglucerase alfa application in process	Yes, given by treating physician and/or nurse to the patient/caregiver
Switzerland	Unsure	No	N/A
United Kingdom [28]	Available, first infusion administered by HCP in hospital. Patients with uneventful infusions must proceed to home therapy (HCP-supported or self-administered). Following a reaction, patients can return to the clinic for their next infusion	Yes, once patient expresses desire the HCP and care team assess competency, risk assessment, and safety. Self-administration discussed at clinic visits	Yes, HCP educates on hygiene, reconstitution, cannulation, administration, and disposal
Unites States of America [20]	Available for velaglucerase alfa	Unsure	N/A

COVID-19, coronavirus disease of 2019; ERT, enzyme replacement therapy; HCP, health care provider; N/A, not available

### Data collection and analyses

Data on patient demographics, study drug administration, drug exposure, and adverse events (AEs) were collected at the time of registry entry via the electronic case report form. All available data to January 7, 2021 (FOS) or July 30, 2021 (GOS) were included in the analysis. AEs (regardless of relationship to treatment) with a start date on or after the first self-administered infusion and no later than the last known date of self-administered infusion were reported as being associated with self-administration. All other AEs (regardless of relationship to treatment) were reported as being associated with HCP-supported infusions.

For self-administrations reported in FOS and GOS, exposure to ERT was calculated from dates of first and last self-administered infusions. For HCP-supported infusions, exposure to ERT was calculated from the date of first infusion to the data extract date or to the date of drug discontinuation in both FOS and GOS. For patients who received HCP-supported infusions followed by self-administering infusions, the HCP-supported infusion exposure to ERT was calculated from date of first infusion to start date of self-administration. The total number of infusions per patient per year was derived from an assumed 26 infusions per year based on every other week dosing.

## Results

### Patient demographics

In total, 232 patients received ≥ 1 self-administered dose of ERT and were included in the self-administration analysis populations: 202 from FOS and 30 from GOS. Of the 202 patients who self-administered agalsidase alfa, 153 (75.7%) were from the Netherlands or the United Kingdom. Of all patients who received agalsidase alfa in the Netherlands and the United Kingdom, 84.6% and 28.3%, respectively, were self-administering infusions. Of the 30 patients who self-administered velaglucerase alfa, 11 (36.7%) were from Israel and 19 (63.3%) were from the United Kingdom. Of all patients who received velaglucerase alfa in Israel and the United Kingdom, 3.6% and 23.2%, respectively, were self-administering infusions. A total of 2233 patients in FOS and 784 patients in GOS received in-clinic/hospital or HCP-supported home infusions only and were included in this analysis as patients who received ERT infusions in an HCP-supported setting. Baseline characteristics of patients self-administering ERT at home and those receiving HCP-supported ERT infusions were generally well balanced across groups, except for age at diagnosis in patients who received velaglucerase alfa, with patients in the self-administration group diagnosed younger than patients in the HCP-supported group (Table 2).

**Table 2:** Patient demographics and characteristics

	Agalsidase alfa		Velaglucerase alfa	
	Self-administered infusion (n = 202)	HCP-supported infusion (n = 2233)^a^	Self-administered infusion (n = 30)	HCP-supported infusion (n = 784)^a^
Sex, n (%)				
Male	97 (48.0)	1006 (45.1)	16 (53.3)	346 (44.1)
Female	105 (52.0)	1227 (54.9)	14 (46.7)	438 (55.9)
Country, n (%)				
Israel	0	6 (0.3)	11 (36.7)	292 (37.2)
Netherlands	55 (27.2)	10 (0.4)	0	0
UK	98 (48.5)	248 (11.1)	19 (63.3)	63 (8.0)
USA	0	12 (0.5)	0	266 (33.9)
Other	49 (24.3)^b^	1957 (87.6)^c^	0	163 (20.8)^d^
Age at FOS/GOS entry				
n	202	2233	30	784
Mean (SD)	39.7 (16.7)	42.1 (16.8)	41.7 (17.7)	40.2 (20.6)
Median (range)	40.3 (2.0, 74.9)	43.5 (0.5, 83.5)	40.5 (10.0, 68.6)	38.7 (0, 89.3)
Age at diagnosis				
n (missing)	189 (13)	2128 (105)	25 (5)	728 (56)
Mean (SD)	31.3 (17.5)	35.9 (18.0)	9.1 (13.5)	20.4 (17.2)
Median (range)	29.0 (0, 67)	37.0 (0, 80)	4.3 (0, 64)	16.8 (0, 85)
Age at first ERT, years				
n	202	2233	30	784
Mean (SD)	39.0 (16.8)	41.5 (16.7)	38.4 (16.7)	36.9 (19.8)
Median (range)	39.9 (2.5, 78.7)	43.1 (2.5, 81.5)	37.3 (8.9, 66.3)	35.7 (0.1, 85.7)
Mean exposure to ERT, years; mean (SD)	10.8 (5.17) 0.9–21.5	8.2 (5.6) 0.0–21.0	9.0 (2.91) 1.1–11.7	7.1 (3.91) 0.0–17.2
Mean time from ERT start to self-administered infusion start, years; mean (SD), range	3.0 (3.53) 0.0–16.4	–	0.8 (2.51) 0.0–10.1	–
Mean age at first self-administered infusion, years; mean (SD), range	42.0 (16.59) 9.7–78.7	–	40.3 (16.42) 9.3–68.2	–
Mean number of self-administered infusions per patient; mean (SD), range	92 (114.69) 0.07–462.55	–	210 (94.69) 0.07–305.38	–
Mean duration of self-administered infusions, years; mean (SD), range	3.5 (4.41) 0.0–17.8	–	8.0 (3.64) 0.0–11.7	–

ERT, enzyme replacement therapy; FOS, Fabry Outcomes Survey; GOS, Gaucher Outcomes Survey; HCP, health care provider; SD, standard deviation

^a^The HCP-supported populations do not include patients who transitioned to self-administration after initiating ERT infusions in a clinic/hospital setting

^b^Argentina (n = 2), Australia (n = 1), Canada (n = 9), Czech Republic (n = 1), Denmark (n = 1), France (n = 6), Germany (n = 20), Italy (n = 3), Slovenia (n = 1), Spain (n = 2), Sweden (n = 1), Switzerland (n = 2)

^c^Argentina (n = 76), Austria (n = 33), Australia (n = 41), Belgium (n = 24), Brazil (n = 32), Canada (n = 162), Czech Republic (n = 48), Denmark (n = 3), Finland (n = 33), France (n = 100), Germany (n = 417), Hungary (n = 10), Italy (n = 171), Japan (n = 462), Portugal (n = 12), Russia (n = 14), Slovenia (n = 9), South Korea (n = 4), Spain (n = 102), Sweden (n = 14), Switzerland (n = 57), Taiwan (n = 133)

^d^Albania (n = 6), Argentina (n = 19), Austria (n = 4), Brazil (n = 1), Canada (n = 15), France (n = 24), Germany (n = 21), Italy (n = 2), Paraguay (n = 19), Poland (n = 7), Russia (n = 25), Spain (n = 20)

The type of Gaucher disease diagnosed was known for all 30 patients self-administering ERT and for 769 patients receiving HCP-supported infusions. Most patients had type I Gaucher disease with similar proportions in both groups (28 [93.3%] patients in the self-administration group and 741 [96.4%] patients in the HCP-supported group). In the self-administration group, no patients had type II Gaucher disease, and 2 (6.7%) patients had type III Gaucher disease. In the HCP-supported group, 2 (0.3%) patients had type II Gaucher disease, and 26 (3.4%) patients had type III Gaucher disease.

### Safety

Data from the FOS registry suggest AEs were proportionally less frequently reported in patients who, after initiating infusion of agalsidase alfa in a clinic/hospital setting, went on to self-administer treatment compared with patients who received HCP-supported infusions of treatment (28.2% vs 57.0%, respectively) (Table 3). However, AE rates per 100 patient-years (100PY) of follow-up were similar between the self-administration and HCP-supported groups (7.99 vs 6.78, respectively). The most frequently reported AEs and serious AEs experienced by patients self-administering agalsidase alfa were cardiac disorders, affecting 19 (9.4%) and 12 (5.9%) patients, respectively. An important point for consideration is that the serious AEs reported are most likely associated with Fabry disease itself rather than the ERT. The drug-related AE rate per 100PY was 1.81 for agalsidase alfa. Drug-related and serious AEs were proportionally less frequently reported with self-administered versus HCP-supported infusions with agalsidase alfa. The frequency of recorded IRRs AEs was lower in patients self-administering (4.5%,) versus patients receiving HCP-supported infusions (13.6%). The most reported IRRs by patients self-administering agalsidase alfa were gastrointestinal disorders, affecting 3 (1.5%) patients, while the most reported IRRs in patients receiving HCP-supported infusions were general disorders and administration site conditions affecting 133 (5.5%) patients.

**Table 3:** Summary of adverse events^a^

	Agalsidase alfa		Velaglucerase alfa	
	Self-administered infusion (n = 202)	HCP-supported infusion (n = 2435)^b^	Self-administered infusion (n = 30)	HCP-supported infusion (n = 814)^b^
Any AE, n (%)	57 (28.2)	1389 (57.0)	12 (40.0)	271 (33.3)
AE rates per 100PY	7.99	6.78	4.97	4.67
Drug-related AEs, n (%)	12 (5.9)	395 (16.2)	1 (3.3)	19 (2.3)
Serious AEs, n (%)	36 (17.8)	876 (36.0)	6 (20.0)	160 (19.7)
Blood and lymphatic system disorders	–	18 (0.7)	–	7 (0.9)
Cardiac disorders	12 (5.9)	315 (12.9)	1 (3.3)	18 (2.2)
Congenital, familial, and genetic disorders	–	21 (0.9)	–	1 (0.1)
Ear and labyrinth disorders	–	29 (1.2)	1 (3.3)	1 (0.1)
Endocrine disorders	–	6 (0.2)	–	1 (0.1)
Eye disorders	–	13 (0.5)	–	3 (0.4)
Gastrointestinal disorders	7 (3.5)	124 (5.1)	–	19 (2.3)
General disorders and administration site conditions	1 (0.5)	139 (5.7)	–	20 (2.5)
Hepatobiliary disorders	1 (0.5)	23 (0.9)	–	7 (0.9)
Immune system disorders	–	13 (0.5)	–	–
Infections and infestations	7 (3.5)	206 (8.5)	2 (6.7)	45 (5.5)
Injury, poisoning and procedural complications	3 (1.5)	115 (4.7)	1 (3.3)	37 (4.5)
Investigations	–	39 (1.6)	–	6 (0.7)
Metabolism/nutrition disorders	–	34 (1.4)	–	2 (0.2)
Musculoskeletal and connective tissue disorders	2 (1.0)	66 (2.7)	1 (3.3)	24 (2.9)
Neoplasms	–	62 (2.5)	1 (3.3)	24 (2.9)
Nervous system disorders	2 (1.0)	254 (10.4)	1 (3.3)	26 (3.2)
Perinatal, puerperium, and pregnancy-related conditions	3 (1.5)	12 (0.5)	1 (3.3)	5 (0.6)
Product issues	–	12 (0.5)	–	2 (0.2)
Psychiatric disorders	2 (1.0)	41 (1.7)	–	6 (0.7)
Renal and urinary disorders	4 (2.0)	125 (5.1)	–	8 (1.0)
Reproductive/breast disorders	2 (1.0)	21 (0.9)	–	8 (1.0)
Respiratory, thoracic, and mediastinal disorders	4 (2.0)	87 (3.6)	–	24 (2.9)
Skin and subcutaneous tissue disorders	1 (0.5)	18 (0.7)	–	4 (0.5)
Social circumstances	–	3 (0.1)	–	–
Surgical/medical procedures	4 (2.0)	16 (0.7)	–	–
Vascular disorders	2 (1.0)	54 (2.2)	–	4 (0.5)
Not coded	–	11 (0.5)	–	2 (0.2)
Drug-related serious AEs, n (%)	2 (1.0)^c^	66 (2.7)	0	1 (0.1)
Cardiac disorders	–	14 (0.6)	–	–
Eye disorders	–	1 (< 0.1)	–	–
Gastrointestinal disorders	–	10 (0.4)	–	–
General disorders and administration site conditions	–	17 (0.7)	–	–
Hepatobiliary disorders	–	1 (< 0.1)	–	–
Immune system disorders	–	2 (0.1)	–	–
Infections and infestations	–	3 (0.1)	–	–
Injury, poisoning and procedural complications	–	8 (0.3)	–	–
Investigations	–	3 (0.1)	–	–
Musculoskeletal and connective tissue disorders	–	6 (0.2)	–	1 (0.1)
Neoplasms	–	1 (< 0.1)	–	–
Nervous system disorders	–	18 (0.7)	–	–
Respiratory, thoracic, and mediastinal disorders	–	7 (0.3)	–	–
Skin and subcutaneous tissue disorders	–	3 (0.1)	–	–
Vascular disorders	–	5 (0.2)	–	–
IRRs, n (%)	9 (4.5)	332 (13.6)	0	13 (1.6)
Cardiac disorders	1 (0.5)	17 (0.7)	–	–
Ear and labyrinth disorders	–	6 (0.2)	–	–
Eye disorders	–	6 (0.2)	–	–
Gastrointestinal disorders	3 (1.5)	55 (2.3)	–	2 (0.2)
General disorders and administration site conditions	1 (0.5)	133 (5.5)	–	4 (0.5)
Hepatobiliary disorders	–	4 (0.2)	–	–
Immune system disorders	–	14 (0.6)	–	–
Infections and infestations	–	3 (0.1)	–	–
Injury, poisoning, and procedural complications	2 (1.0)	69 (2.8)	–	6 (0.7)
Investigations	–	37 (1.5)	–	–
Metabolism/nutrition disorders	–	3 (0.1)	–	–
Musculoskeletal and connective tissue disorders	1 (0.5)	30 (1.2)	–	3 (0.4)
Neoplasms	–	1 (< 0.1)	–	–
Nervous system disorders		59 (2.4)	–	1 (0.1)
Psychiatric disorders	–	3 (0.1)	–	–
Renal and urinary disorders	–	11 (0.5)	–	–
Respiratory, thoracic, and mediastinal disorders	1 (0.5)	37 (1.5)	–	2 (0.2)
Skin and subcutaneous tissue disorders	1 (0.5)	50 (2.1)	–	–
Vascular disorders	1 (0.5)	38 (1.6)	–	–
Not coded	–	3 (0.1)	–	–
Fatal AEs, n (%)	0	193 (7.9)^d^	1 (3.3)^e^	31 (3.8)^f^
Mean time from start of drug to first treatment-emergent event^g^, years				
Non-serious AE	2.6	2.9	4.6	4.9
Serious AE	3.0	4.2	4.5	4.7
IRR	2.9	2.1	–	2.6

AE, adverse event; ERT, enzyme replacement therapy; HCP, health care provider; IRR, infusion-related reaction; PY, patient years

^a^AEs were classified according to terminology used in the MedDRA dictionary version 23 and listed by system organ class

^b^The HCP-supported populations included all patients who received ≥1 ERT infusion in a clinic/hospital setting or HCP-supported infusion at home, including those patients who later transitioned to self-administration and self-administered ≥1 ERT infusion

^c^One patient had two reported drug-related serious AEs of paranoia and abnormal behavior, and another patient had one reported drug-related serious AE of myocardial infarction with agalsidase alfa

^d^Of these, 5 patient deaths were considered as possibly related to agalsidase alfa (septic shock, lung edema and heart failure, cardiac failure acute/pneumonia, sudden death, and arrhythmia)

^e^One patient (age 64) died due to cardiac arrest after 8.2 years of self-administering infusions. This death was not considered to be related to velaglucerase alfa

^f^No patient deaths were considered related to velaglucerase alfa

^g^Refers to mean time from start of drug by each administration methods (self-administered vs HCP-supported)

In the self-administration population, one patient had two reported drug-related serious AEs of paranoia and abnormal behavior, and another patient had one reported drug-related serious AE of myocardial infarction. No AEs in the self-administration group led to discontinuation whereas 30 (1.2%) patients in the HCP-supported group discontinued agalsidase alfa infusions. No patients died from AEs in the self-administration group, while 193 (7.9%) patients died from serious AEs (not related to the infusion) in the group receiving HCP-supported infusions. Of these, 5 (0.2%) patient deaths were possibly related to agalsidase alfa, and the remaining 188 deaths were considered unrelated to treatment. The possibly treatment-related but not IRRs fatal AEs (8 events) were abnormal hepatic function, acute cardiac failure, arrhythmia, cerebral infarction, decreased platelet count, pulmonary oedema, septic shock, and sudden death. The mean times from starting self-administration to the first treatment-emergent AE (regardless of relationship to treatment), serious AE (regardless of relationship to treatment), and IRR were 2.6 years, 3.0 years, and 2.9 years, respectively. For HCP-supported infusions, the mean times from starting ERT to first non-serious AE, serious AE, and IRR were 2.9 years, 4.2 years, and 2.1 years, respectively.

Data from the GOS registry suggest the proportion of patients who reported any AE with velaglucerase alfa self-administration was slightly higher compared with patients who received HCP-supported infusions (40.0% vs 33.3%, respectively). However, AE rates per 100PY were similar between groups (4.97 vs 4.67, respectively). Lower or similar proportions of patients experienced drug-related, serious, or fatal AEs, or IRRs (Table 3), with a drug-related AE rate of 0.22 per 100PY for velaglucerase alfa. The most frequently reported AEs and serious AEs experienced by patients self-administering velaglucerase alfa were skin and subcutaneous disorders (4 [13.3%] patients) and infections (2 [6.7%] patients), respectively. Patients self-administering velaglucerase alfa reported no IRRs versus 1.6% of patients receiving HCP-supported infusions. The most reported IRRs in patients receiving HCP-supported infusions, were injury, poisoning, and procedural complications, affecting 6 (0.7%) patients.

In the self-administration group, one (3.3%) patient died from cardiac arrest deemed unrelated to velaglucerase alfa after 8.2 years of self-administering infusions. In the group receiving HCP-supported infusions, 31 (3.8%) patients died from AEs, and none were considered related to velaglucerase alfa. No AEs in the self-administration group led to discontinuation, whereas 5 (0.6%) patients in the HCP-supported group discontinued velaglucerase alfa infusions. The mean times from starting self-administration to first treatment-emergent AE (regardless of relationship to treatment) and serious AE (regardless of relationship to treatment) were 4.6 years and 4.5 years, respectively. For HCP-supported infusions, the mean times from starting ERT to first non-serious AE, serious AE, and IRR were 4.9 years, 4.7 years, and 2.6 years, respectively.

## Discussion

The results of this analysis from the FOS and GOS registries support that there are no additional safety concerns with self-administration of the ERTs agalsidase alfa or velaglucerase alfa versus HCP-supported infusions. The frequency of IRRs and drug-related AEs was similar or lower in the self-administration groups compared with those receiving HCP-supported infusions. The proportion of patients self-administering treatment who had AEs was relatively low, and the overall proportion of patients reporting serious AEs was lower with self-administration of ERT versus receiving HCP-supported ERT infusions. This may be attributed to a combination of factors, including the return of patients who experience an IRR or AE to receiving HCP-supported infusions in the clinic/hospital setting, switching to self-administration after no observed IRRs or AEs with HCP-supported infusions, the high familiarity of patients with these ERTs, and their favorable safety profiles. Drug-related serious AEs in patients self-administering ERT were infrequent, with a total of three events reported as drug-related by two patients self-administering agalsidase alfa (paranoia and abnormal behavior in one patient, and myocardial infarction in another patient) and none reported by patients self-administering velaglucerase alfa.

The follow-up period for patients self-administering ERTs was shorter and hence AEs were recorded over a shorter time-period compared with follow-up time for AEs in patients receiving HCP-supported infusions. However, adjusting the AE rates per 100PY for both agalsidase alfa and velaglucerase alfa resulted in similar rates between the self-administration and HCP-supported infusion populations, indicating that the frequency of AEs was similar in both groups. The long mean time to first treatment-emergent AE from the start of self-administration of both agalsidase alfa and velaglucerase alfa suggests that AEs are not likely to arise from inadequate training or poor infusion practices. Further, patients self-administering agalsidase alfa and velaglucerase alfa have been doing so successfully over a number of years with both ERTs.

Comparisons of patients self-administering ERT infusions with patients receiving HCP-supported infusions need to be interpreted with caution owing to certain differences between these populations. Firstly, all patients start treatment in the clinic/hospital and self-administration programs are recommended only for patients who have been shown to tolerate HCP-supported infusions well. Secondly, before transitioning to self-administration and under the responsibility of the treating physician, patients must complete the required number of HCP-supported infusions, which varies between countries. Thirdly, AEs were recorded over a shorter time-period for patients self-administering infusions than for those receiving HCP-supported infusions. Nonetheless, the results of our analysis suggest that self-administration of agalsidase alfa and velaglucerase alfa has a favorable safety profile and can be a suitable option for many patients. Indeed, this evaluation of data from the FOS and GOS patient registries revealed that self-administration of ERT is already being practiced successfully in several countries worldwide.

Home administration by HCPs of ERT with agalsidase alfa or velaglucerase alfa has been shown to improve satisfaction, convenience, and QoL of patients [30, 31]. Additionally, ERT home administration frees up time and space in the clinic, which is of benefit to HCPs. Further, transitioning to self-administration of ERT affords both patients and HCPs the convenience of not traveling to and from the patient’s home, cost-saving advantages and reduces the impact of travel on the environment overall contributing to treatment sustainability. Self-administration practices, where agalsidase alfa and velaglucerase alfa infusions are managed by the patient, provide independence and control to choose a time of administration that is convenient. Further, self-administering infusions allows patients to contribute to and manage their own care. Notably, during the COVID-19 pandemic, patients who were already self-administering ERT did not have to stop infusions or have their regimen disrupted; they benefited from being able to continue treatment in the comfort and safety of their own home, without the risk of exposure to HCP personnel [32, 39].

This study had some limitations that should be considered. Owing to the collection of real-world data and the voluntary nature of participation in the FOS and GOS registries, data may be incomplete, underreported, or subject to selection bias. For example, underreporting of AEs and IRRs may be more likely with patients self-administering ERT infusions and managing their own care compared with infusions being administered and monitored by an HCP in the clinic/hospital or home setting. Moreover, the number of patients included in this analysis is relatively small, particularly with respect to the velaglucerase alfa self-administration population. Lastly, the selection of patients for self-administration, self-administration practices, and patient training may vary between sites and countries. Exploring if any country-specific practices are associated with improved outcomes could help enhance the care of patients who are self-administering ERT infusions. Countries not yet implementing self-administration practices could benefit from guidance on best practices provided by other countries with established self-administration programs.

## Conclusions

These findings suggest that self-administration of agalsidase alfa or velaglucerase alfa infusions are not associated with additional safety risks compared with HCP-supported infusions and are a suitable option for qualifying patients. Information relating to self-administration of ERT may help to inform treatment management decisions as part of individualized patient care. Patients self-administering ERT infusions are afforded benefits including independence, flexibility, and control, all of which encourage self-management of their care. Successful self-administration programs are established in several countries and could serve as resources for other countries considering implementing self-administration practices for agalsidase alfa and velaglucerase alfa. Further research is warranted to support these findings and to explore further the long-term safety and efficacy of ERT self-administration.

## Data Availability

The datasets, including the redacted study protocol, redacted statistical analysis plan, and individual Participants data supporting the conclusions of this article, will be made available after the publication of study results within three months from initial request to researchers who provide a methodologically sound proposal. The data will be provided after its de-identification, in compliance with applicable privacy laws, data protections and requirements for consent and anonymization.

## Abbreviations

AE: Adverse event
COVID-19: Coronavirus disease of 2019
ERT: Enzyme replacement therapy
FOS: Fabry Outcome Survey
GOS: Gaucher Outcome Survey
HCP: Health care provider
ICF: Informed consent form
ICH E6: International Conference on Harmonisation Good Clinical Practice guidelines, sixth edition
IRR: Infusion-related reaction
PY: Patient-years
SD: Standard deviation
